# Design and rationale of the comprehensive evaluation of risk factors in older patients with AMI (SILVER-AMI) study

**DOI:** 10.1186/s12913-014-0506-4

**Published:** 2014-11-05

**Authors:** John A Dodson, Mary Geda, Harlan M Krumholz, Nancy Lorenze, Terrence E Murphy, Heather G Allore, Peter Charpentier, Sui W Tsang, Denise Acampora, Mary E Tinetti, Thomas M Gill, Sarwat I Chaudhry

**Affiliations:** Leon H Charney Division of Cardiology, Department of Medicine, New York University School of Medicine, New York, NY USA; Section of Geriatrics, Department of Internal Medicine, Yale University School of Medicine, New Haven, CT USA; Section of Cardiovascular Medicine, Department of Internal Medicine, Yale University School of Medicine, New Haven, CT USA; Center for Outcomes Research and Evaluation, Yale-New Haven Hospital, New Haven, CT USA; Robert Wood Johnson Foundation Clinical Scholars Program, Yale University School of Medicine, New Haven, CT USA; Department of Health Policy and Administration, Yale School of Public Health, New Haven, CT USA; Section of General Internal Medicine, Yale University School of Medicine, Harkness Office Building, Room 411, New Haven, CT 06520 USA

**Keywords:** Acute myocardial infarction, Aging, Hospital readmission, Health status

## Abstract

**Background:**

While older adults (age 75 and over) represent a large and growing proportion of patients with acute myocardial infarction (AMI), they have traditionally been under-represented in cardiovascular studies. Although chronological age confers an increased risk for adverse outcomes, our current understanding of the heterogeneity of this risk is limited. The Comprehensive Evaluation of Risk Factors in Older Patients with AMI (SILVER-AMI) study was designed to address this gap in knowledge by evaluating risk factors (including geriatric impairments, such as muscle weakness and cognitive impairments) for hospital readmission, mortality, and health status decline among older adults hospitalized for AMI.

**Methods/Design:**

SILVER-AMI is a prospective cohort study that is enrolling 3000 older adults hospitalized for AMI from a recruitment network of approximately 70 community and academic hospitals across the United States. Participants undergo a comprehensive in-hospital assessment that includes clinical characteristics, geriatric impairments, and health status measures. Detailed medical record abstraction complements the assessment with diagnostic study results, in-hospital procedures, and medications. Participants are subsequently followed for six months to determine hospital readmission, mortality, and health status decline. Multivariable regression will be used to develop risk models for these three outcomes.

**Discussion:**

SILVER-AMI will fill critical gaps in our understanding of AMI in older patients. By incorporating geriatric impairments into our understanding of post-AMI outcomes, we aim to create a more personalized assessment of risk and identify potential targets for interventions.

**Trial registration:**

Trial registration number: NCT01755052.

## Background

Among patients hospitalized for acute myocardial infarction (AMI), one-third are 75 years or older [1]. The number of incident AMI cases in this age group is expected to double over the next 30 years as the mean age of the general population increases [2], yet this group has been under-represented in AMI clinical trials and epidemiologic studies [3]. This group is fundamentally different from younger patients with AMI: they have a higher burden of comorbid diseases and aging-related physical and cognitive impairments, as well as lower physiologic reserve. Results from studies of younger patients with AMI may therefore not be directly applicable to this growing population.

While advanced age is a risk factor for adverse outcomes after AMI [1], there is great heterogeneity among older patients and chronological age is a relatively crude indicator of physiological age [3,4]. Despite emerging interest in understanding the role of geriatric conditions (such as physical and cognitive impairments) as they pertain to cardiovascular outcomes [3], the majority of studies examining risk have used administrative datasets that lack this information [5], and none of the currently available risk stratification tools for AMI incorporate geriatric conditions. Furthermore, these risk models have only modest discrimination in older patients [6,7], and were designed solely to predict clinical events (i.e., mortality, reinfarction) rather than patient-centered outcomes, such as health status.

**Figure 1:**
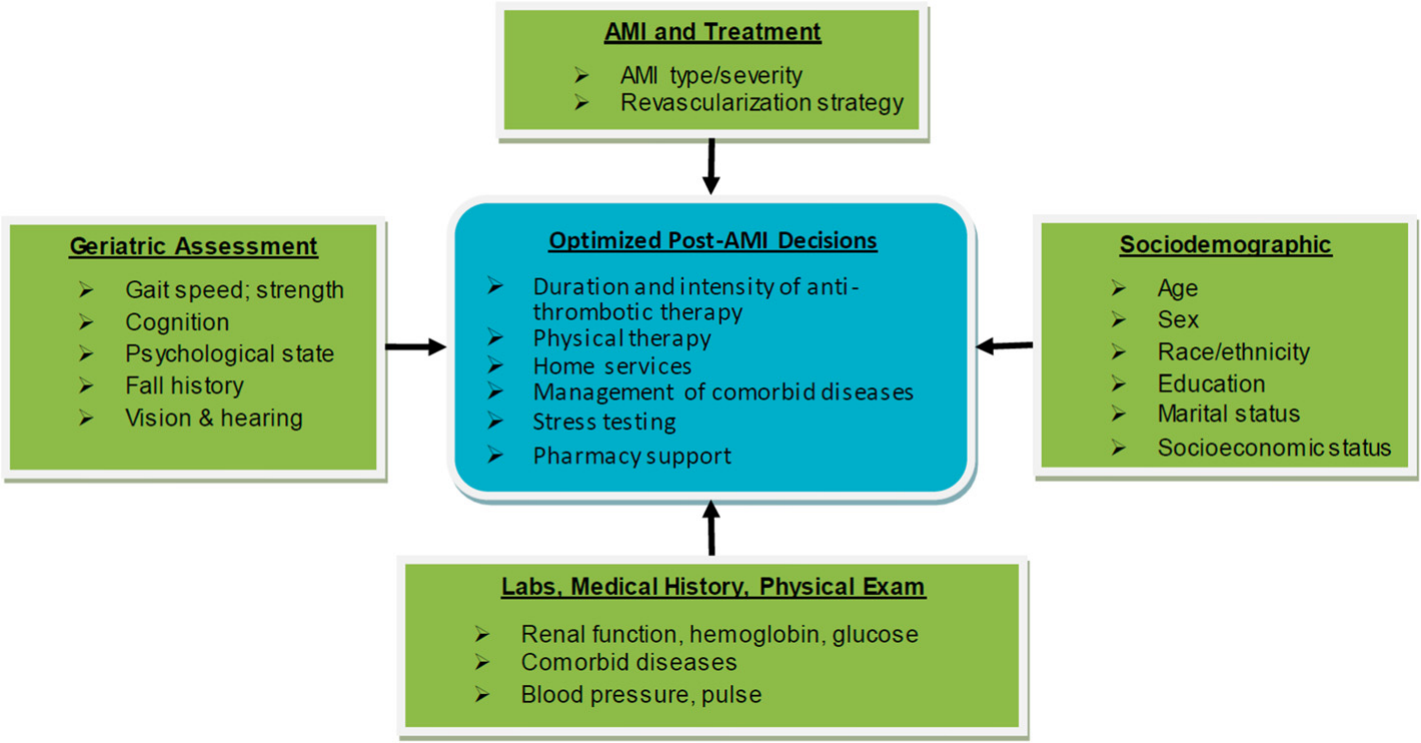
**Comprehensive assessment to inform post-AMI decision making in older adults.** Assessment of multiple domains may optimize medical decision-making. (For clarity, not all possible risk factors or post-AMI clinical decisions are listed).

The Comprehensive Evaluation of Risk Factors in Older Patients with AMI (SILVER-AMI, R01HL115295, PI Chaudhry) study was designed to address these knowledge gaps by melding principles from geriatrics and cardiology with the goal of more precisely characterizing the risk of traditional clinical events, as well as patient-centered outcomes, in older adults with AMI. The primary objective is to generate risk models that predict all-cause readmission, all-cause mortality, and decline in health status, that exceed the capabilities of currently available risk models in older adults. We hypothesize that the incorporation of geriatric conditions will lead to better discrimination than existing risk models. Secondary objectives are to: (1) estimate the frequency and determinants of adverse post-hospital events important in this population, including medication side effects, symptoms, falls, and bleeding; and (2) describe AMI processes of care (e.g. door-to-balloon time, revascularization strategies, use of secondary prevention medications). We believe that achieving these objectives can inform complex post-AMI decision-making in older adults (Figure 1). The purpose of this article is to describe the design, rationale, and methods of the SILVER-AMI study.

## Methods/Design

SILVER-AMI is a multi-center, longitudinal cohort study in which participants undergo a comprehensive baseline (in-hospital) assessment, and then complete a follow-up telephone interview (six months after discharge). The study received approval from the Yale Institutional Review Board (IRB), as well as IRBs at the participating study sites, and is registered at www.clinicaltrials.gov (NCT01755052, registered 11/27/2012).

### Study sites: management and training

**Figure 2:**
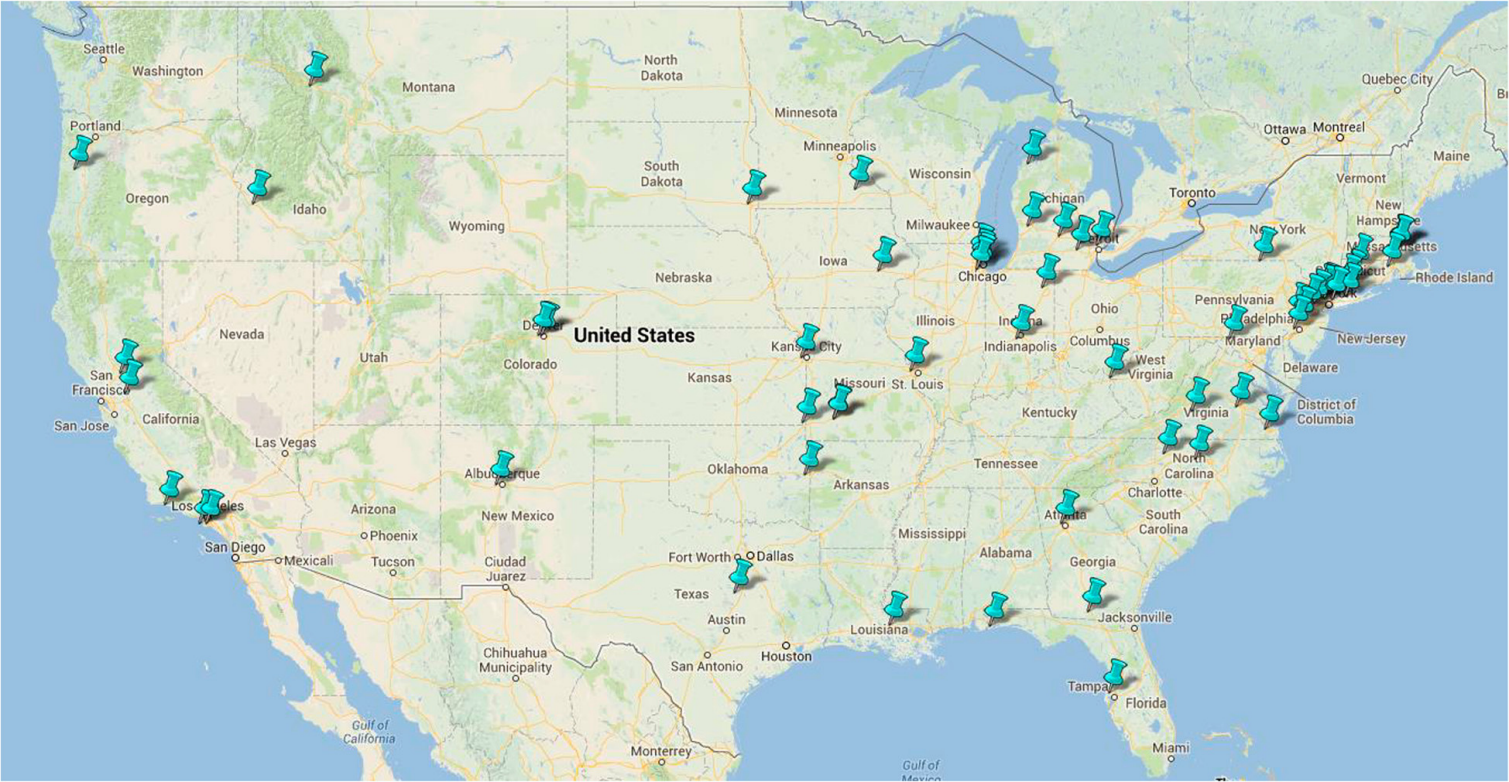
**SILVER-AMI national recruitment network.**

The recruitment network for SILVER-AMI was assembled with the goal of reflecting a wide geographic distribution and a diverse mix of practice types. There are approximately 70 recruitment sites located in the United States representing 27 states (Figure 2). The SILVER-AMI network is built upon the foundation of the recruitment network assembled for the VIRGO study [8] and additional sites were recruited through the American College of Cardiology’s Section of Geriatric Cardiology. The majority of sites (71.4%) are non-academic hospitals. Hospitals are heterogeneous in size (range: 83 to 2292 beds, median 475) and location (urban: 47.1%, suburban/rural: 52.9%).

Each site identifies a site research coordinator who has primary responsibility for implementing the study protocol. Prior to study initiation, the site research coordinators complete required trainings (in the recruitment of older hospitalized patients, obtaining of informed consent, and use of the electronic data capture system) conducted by the Yale Coordinating Center, and are supplied with standardized equipment to perform the physical assessments.

### Patient screening and determination of eligibility

Screening is performed in the hospital setting by the site research coordinators who review daily admission records to identify potentially eligible participants. Site research coordinators then perform a medical record review to confirm eligibility in accordance with the criteria found in the Third Universal Definition of Myocardial Infarction [9], listed in Table 1.

**Table 1:** **Eligibility Criteria for SILVER-AMI**

**Inclusion criteria**	**Exclusion criteria**
Age ≥75 years	Initial troponin elevation occurs >24 hours after hospital admission
Diagnosis of acute myocardial infarction	Acute myocardial infarction secondary to inpatient procedure or surgery
- Serum troponin I or troponin T elevation above laboratory’s upper limit of normal, and either (1) ischemic ECG findings, (2) anginal symptoms, (3) imaging evidence of new loss of viable myocardium or new regional wall motion abnormality; (4) identification of an intracoronary thrombus on angiography^a^	Transferred from another hospital with a length of stay >24 hours at the referring hospital
	Incarcerated
	Unable to provide informed consent with no proxy available

^a^In accordance with Universal Definition of Myocardial Infarction [14].

If, during the screening process, the site research coordinator is concerned about the potential participant’s decisional capacity, the University of California, San Diego Brief Assessment of Capacity to Consent (UBACC) is administered [10]. If potential participants demonstrate impaired decision-making ability based on the UBACC, proxy consent is obtained in compliance with local regulations for legally authorized representatives.

In order to confirm study eligibility and provide further classification, medical records from enrolled participants are adjudicated by two physician investigators (JD, SC) at the Yale Coordinating Center, who confirm the baseline AMI diagnosis, interpret the diagnostic electrocardiogram (ECG), and determine whether the event represents ST elevation myocardial infarction (STEMI) or non ST elevation myocardial infarction (NSTEMI) based on previously published criteria [9].

### In-hospital assessment

Participants undergo an interview and physical assessment by the site research coordinator during their baseline AMI hospitalization (Table 2). In addition to sociodemographic and presentation characteristics, specific domains are tested which are described below. Participants receive a $25 gift card for completing this assessment.

**Table 2:** **Domains and timing of assessments in SILVER-AMI**

**Domain (Instruments)**	**Baseline interview**	**Medical record abstraction**	**Six-month interview**	**Six-month event review**
Sociodemographic characteristics (race, marital status, education, income)	x			
Clinical characteristics				
Presenting symptoms	x	x		
Cardiac history		x		
Non-cardiac comorbidities		x		
In-hospital medications		x		
Procedures (cardiac catheterization, PCI, CABG, pacemaker, ICD)		x		x
In-hospital cardiac testing (echo, stress test)		x		
In-hospital complications		x		
Discharge medications		x		
Discharge disposition		x		
Health status measures, symptom burden, psychosocial assessments				
General health (SF-12)	x		x	
CAD-specific health (SAQ-7)	x		x	
General symptoms (ESAS)	x		x	
Social support (MOS-SSS)	x			
Comprehensive geriatric assessment				
Depressive symptoms (PHQ-8)	x		x	
Cognition (TICS, COWAT)	x			
Vision and hearing	x			
Grip strength (dynamometer)	x			
Functional mobility (TGG)	x			
Activities of Daily Living	x		x	
Fall assessment	x		x	
Delirium (abbreviated CAM)	x		x	
Orthostatic vital signs	x			
Alcohol and tobacco frequency	x			
Post-discharge health care utilization				
Emergency Department visits				x
Hospital readmissions				x
Cardiac procedures				x
Medication adverse effects				x
Medication adherence				x
Deaths				x

*Abbreviations:* *PCI* percutaneous coronary intervention, *CABG* coronary artery bypass grafting, *ICD* implantable cardioverter defibrillator, *SF-12* Short-Form 12, *CAD* coronary artery disease, *SAQ-7* abbreviated Seattle Angina Questionnaire, *ESAS* Edmonton Symptom Assessment Scale, *MOS-SSS* Medical Outcomes Study Social Support Scale, *PHQ-8* Patient Health Questionnaire, *TICS* Modified Telephone Interview for Cognitive Status, *COWAT* Controlled Word Association Test, *TGG* Timed Get Up and Go, *CAM* Confusion Assessment Method.

#### Health status measures, symptom burden, and psychosocial assessments

Participants are asked about health status with the Short Form 12 (SF-12) [11], a widely used health status measure that has demonstrated reliability and validity in many populations, including older adults. In addition, dimensions of health status specific to coronary artery disease are assessed with the abbreviated Seattle Angina Questionnaire (SAQ) [12] which measures three angina-related domains: physical limitation, angina frequency, and quality of life.

Symptoms are assessed with the Edmonton Symptom Assessment Scale (ESAS) [13], a visual analogue scale that contains nine common symptoms (pain, fatigue, nausea, depression, anxiety, drowsiness, appetite, well-being, shortness of breath) that are rated by participants on a severity scale of 1 (no symptoms) to 10 (worst possible symptoms).

Depression is known to be prognostically important in patients with cardiovascular disease; [14] we therefore assess depressive symptoms with the eight-item Patient Health Questionnaire (PHQ-8) [15]. In addition, a shortened five-item version of the Medical Outcomes Study Social Support Scale (MOS-SSS) evaluates perceived social support [16].

#### Geriatric conditions

General cognitive function is evaluated with the Telephone Interview of Cognitive Status (TICS) [17]. We chose the TICS because it can be administered in <10 minutes, can be used to detect mild as well as severe cognitive impairments, does not require writing (i.e. can be used despite visual or motor impairments), and can be converted to a Mini Mental State Examination (MMSE) equivalent [18]. In addition to general cognitive function, we include an assessment of executive function, which is relevant for specific tasks such as adherence to complex medical regimens after discharge, with the Controlled Word Association Test (COWAT) [19].

Vision questions are adapted from the Visual Functioning Questionnaire (VFQ-25); [20] participants are asked to rate their general vision on an ordinal scale (from “excellent” to “very poor”), and to indicate whether they have difficulty reading print or doing work or hobbies. Hearing is assessed with a global question (“do you have a hearing problem now?”) that has shown good sensitivity and specificity compared with audiography [21].

Muscle strength and functional mobility are important predictors of future disability and health status decline [22,23]. For muscle strength we chose to measure grip strength, which is assessed by the participant using their dominant hand to squeeze a handheld dynamometer (B&L Engineering, Santa Ana, CA); results are recorded in kilograms (kg) for three successive attempts. Functional mobility is measured with the Timed Get Up and Go (TUG) test [23], which requires the participant to rise from a seated position, walk 3 meters, and then return to the chair and sit down. The time it takes to complete this test in seconds is recorded, as well as a subjective rating of the person’s movements on a scale of 1–5 from normal to severely abnormal.

Activities of Daily Living (ADLs) [24] are measured with four interview items that assess whether participants are able to perform, without help from another person, the tasks of bathing, dressing, getting out of a chair, and ambulating. Participants are also asked how many falls they have had in the past year (from none to ≥ four).

### Medical record abstraction

**Figure 3:**
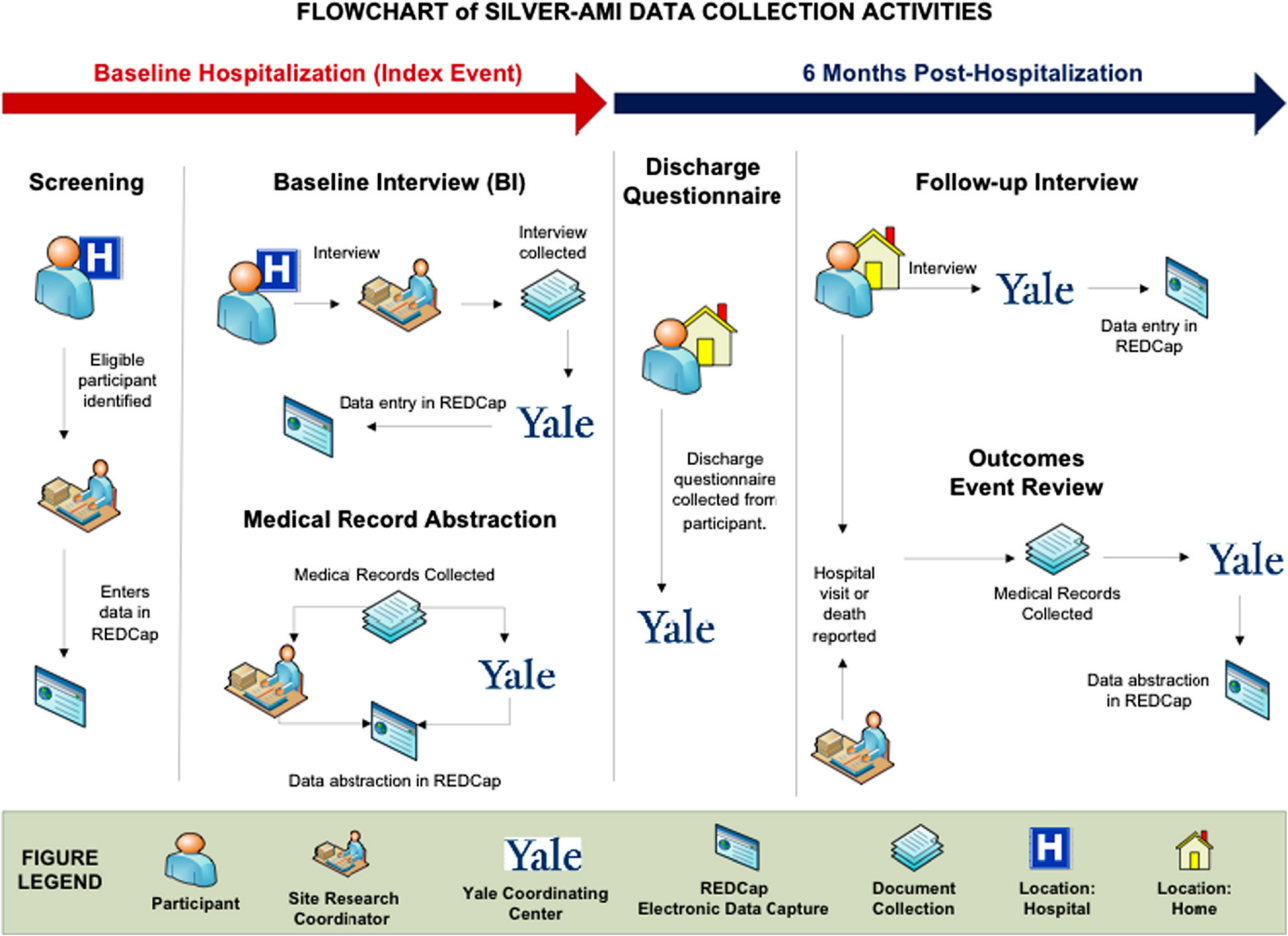
**Procedures for data collection and management in the SILVER-AMI study.** Data collection and management involves a coordinated effort between study sites and Yale Coordinating Center.

To complement the participant interview and assessment, an in-depth medical record review is performed by the site research coordinator who collects information about clinical status at the time of the initial presentation (blood pressure, heart rate, presence of decompensated heart failure), comorbidities, laboratory results, in-hospital adverse events, and discharge disposition (Figure 3). Medical records are also provided to the Yale Coordinating Center where a research nurse (NL) conducts an in-depth review to collect information about medications, cardiac procedures, and discharge instructions.

### Six-month interview

The six-month telephone interview is conducted by staff at the Yale Coordinating Center. For participants with impaired decisional capacity at baseline, the six-month interview is completed with both the participant and assigned proxy.

The six-month interview repeats several key components of the baseline interview (Table 2), including questions about both general and disease-specific health status. In addition to the outcomes of re-admission and death, we define a decline in health status as a 10% reduction in overall quality of life as measured by the SF-12 [25]. Participants are also questioned on whether they have been re-hospitalized, and whether they have experienced any signs or symptoms that they attribute to medications (i.e., “adverse effects”) since their AMI discharge. If present, the participant is asked the medication to which they attribute the symptoms and if any action was taken to address the adverse effect. Participants receive an additional $50 gift card for completing the six-month interview.

### Events review

At the six-month time point, the site research coordinator collects medical records on any hospital readmissions, outpatient cardiac procedures, emergency department (ED) visits, and deaths from the index hospital, as well as all other hospitals the participant reported using at the time of baseline interview. Medical records are provided to the Yale Coordinating Center where the events are reconciled with the participant’s self-reported hospitalizations during the 6-month phone interview. Any outstanding records are then collected. Medical records and death certificates are reviewed by physician investigators to determine whether the reported events represent true hospital admissions, as well as the primary discharge diagnosis, and/or cause of death.

### Data management

The baseline interview is completed by the site research coordinator and transmitted electronically to the Yale Coordinating Center within three days (Figure 3). A Yale research assistant reviews the form and queries the site for missing or incomplete items. The site also transmits medical records, including emergency department and inpatient notes, ECGs, cardiac catheterization and operative (CABG) reports, and echocardiogram results, for abstraction by a research nurse at the Yale Coordinating Center.

All study data are managed at the Yale Coordinating Center using REDCap, an NIH-supported, HIPAA-compliant web-based Electronic Data Capture (EDC) system [26]. A custom Site Portal website manages the various administrative and scientific site workflows, including: site enrollment, IRB tracking; study document distribution, staff training, participant enrollment, invoicing, and site performance monitoring. In addition, we have integrated REDCap’s error detection and resolution features into a Data Query Quality Control system (DQCQ) that allows for error checks of varying complexity using SAS programs.

### Statistical analysis

The primary outcomes of SILVER-AMI are the occurrence of all-cause hospital readmission, all-cause death, and decline in health status within six months of hospital discharge. Using independent risk factors that include cardio-centric factors (e.g. STEMI vs. NSTEMI, systolic blood pressure, Killip Class), as well as geriatric impairments, we will construct risk stratification models that predict the probability of each outcome for specific combinations of the risk factors. These models will be variants of the Cox regression models for the time-to-event outcomes (hospital readmission and mortality) and the logistic model used for decline in health status. Because mortality serves as a competing risk for hospital readmission, we will evaluate the associations of explanatory variables with both time-to-event outcomes using the competing risk approach of Fine and Gray [27]. All linearity assumptions will be checked graphically and deviations from model additivity will be accommodated by an examination of all statistically significant, clinically indicated two-factor interactions. All missing data will be examined for the nature of missingness, i.e., whether missing at random, and if justifiable, multiple imputation will be applied. Models will be evaluated with an examination of residuals and goodness-of-fit statistics using SAS/STAT ® V9.3 (SAS Institute, Cary, NC) or later, with statistical significance defined as a two-sided p-value <0.05.

Internal and external validation techniques will then be used to test the reproducibility of the risk stratification tools. For internal validation, jackknife methods [28] will be used to evaluate the percent of correct classification; external validation will be performed on the 500 randomly selected participants who were excluded from both model building and internal validation.

Once the final models have been developed, user-interface applications will be developed for “smart-phones” and other hand-held devices. The model coefficients for each outcome will be stored in hand-held applications whose outputs will provide the probability of each outcome. The inputs to the applications will consist of simple yes/no entries denoting the presence or absence of risk factors. Calculation will be presented in real time, and the results will list specific risk factors, and probability of the outcomes.

### Sample size and power

Of the SILVER-AMI cohort of 3000 participants, 2500 participants will serve as the development cohort and 500 will be randomly selected as the validation cohort. Based on 2008 Medicare data, we posited outcome rates of 39% and 28% for all-cause readmission and mortality. Based on previous research [29], we also assumed that a 10% decline in health status, as measured by the SF-36, would occur in 35% of surviving participants. With regard to the explanatory variables of primary interest, i.e., geriatric conditions and socio-demographic and clinical factors, we assumed each would exhibit measures of relative risk ≥1.3 for each time-to-event outcome and ≥1.5 for decline in health status. The 2500 participants in the development cohort provide 90% power to detect the stated magnitudes of relative risk for prevalence of the explanatory variables between 30% and 50%. For the validation sample, the accepted standard for time-to-event and logistic models should be large enough to contain a minimum of 100 outcome events [30]. For mortality, the least frequently expected outcome, the additional sample of 500 should allow for 140 events. The validation size of 500 will be preserved regardless of overall mortality and dropout.

## Discussion

SILVER-AMI will fill critical gaps in our understanding of AMI in older patients. Using a large, diverse cohort of older patients cared for in a variety of community-based and academic settings, we will evaluate determinants of post AMI risk (for readmission, mortality, and health status decline). Secondarily, we will describe patterns of adverse post-hospital events such as falls, bleeding, and medication adverse effects.

The SILVER-AMI cohort builds on a long tradition of NHLBI-funded longitudinal cohort studies that have described care patterns and optimal management strategies in patients with AMI [31-33]. Several unique aspects of our study will extend knowledge beyond that provided in earlier cohorts. First, our study uses a comprehensive geriatric assessment, which includes an evaluation of an individual’s cognition, muscle strength, physical function, depressive symptoms, and falls history. While this assessment has been increasingly incorporated into the care of patients with cancer and has been shown to enhance prediction of outcomes [34], its use in the care of patients with cardiovascular disease is still uncommon. In addition to creating a more personalized assessment of risk, the geriatric assessment may identify specific impairments that can serve as targets for interventions. For example, patients with cognitive impairments can receive extra assistance with managing medications and follow-up appointments; in patients at high risk for medication side effects, caution may be needed when considering long-term dual antiplatelet therapy, oral anticoagulants, or beta blockers.

A second unique feature of SILVER AMI involves the incorporation of patient-centered outcomes not available in most prior AMI registries, including symptoms, health status, angina-specific quality of life, and adverse effects from cardiovascular medications. Studies repeatedly show that older patients may value these outcomes in decision-making more importantly than simply prolonging life [35]. It is therefore important to understand health status in the context of AMI, as well as which patients achieve measurable improvement in health status after interventions, such as PCI and CABG.

Third, there are several specific products that will be developed from the data collected in SILVER-AMI. These include separate risk models for three outcomes (readmission, mortality, and decline in health status) that, once created, will be adapted for PC-based and smartphone use, similar to the Framingham Risk Calculator [36] or TIMI Risk Score [37]. Such decision aids may help to identify patients in need of resource intensive post-hospital interventions and therefore lead to improvements in real-time decision making at the point of care.

Finally, the recruitment network for SILVER-AMI includes approximately 70 sites across the U.S. in a variety of geographic settings. The network involves a large range of hospital sizes, as well as a mixture of academic and community hospitals, ensuring a diverse mixture of patients and practice patterns that will lend external validity to our findings. This network also has the potential to be used in future epidemiologic studies of other cardiac conditions, or for trials of interventions aimed at improving outcomes among older adults.

## Conclusions

With the aging of the population, it is imperative to identify determinants of post-AMI outcomes in older adults, and to understand contemporary practice patterns in the care of this population. Subsequently, the knowledge gained from the SILVER-AMI cohort may be used to design interventions directed at reducing hospital readmission and mortality and improving health status.

## Abbreviations

AMI: Acute myocardial infarction
ECG: Electrocardiogram
STEMI: ST elevation myocardial infarction
NSTEMI: Non ST elevation myocardial infarction
SF-12: Short Form 12
SAQ: Seattle Angina Questionnaire
ESAS: Edmonton Symptom Assessment Scale
MOS-SSS: Medical Outcomes Study Social Support Scale
TICS: Telephone Interview of Cognitive Status
MMSE: Mini Mental State Examination
COWAT: Controlled Word Association Test
VFQ-25: Visual Functioning Questionnaire
TUG: Timed Get Up and Go
ADLs: Activities of Daily Living
ED: Emergency department
CABG: Cardiac catheterization and operative
EDC: Electronic Data Capture
DQCQ: Data Query Quality Control system
NIH: National Institutes of Health
NHLBI: National Heart, Lung, and Blood Institute
NIA: National Institute of Aging
